# The Impact of Social Stress and Healthy Lifestyle on the Mortality of Chinese Older Adults: Prospective Cohort Study

**DOI:** 10.2196/75942

**Published:** 2025-08-12

**Authors:** Jin Yang, Jilong Huang, Qingmei Huang, Jian Gao, Dan Liu, Zhihao Li, Yuebin Lv, Xiaoming Shi, Chen Mao

**Affiliations:** 1 Department of Epidemiology, School of Public Health, Southern Medical University, No. 1023-1063, Shatai South Road, Baiyun District, Guangzhou, Guangdong, 510515, China, 86 2061648430; 2 China CDC Key Laboratory of Environment and Population Health, National Institute of Environmental Health,, Chinese Center for Disease Control and Prevention, Beijing, China; 3 National Institute of Health Data Science of China, Southern Medical University, Guangzhou, Guangdong, China

**Keywords:** social stress, healthy lifestyle, mortality, older adults, cohort study

## Abstract

**Background:**

With social progress, social stress (SS) has become a key factor affecting health. Unhealthy lifestyles may exacerbate these effects. However, the relationship between SS, lifestyle, and older adults’ mortality rate still needs to be studied.

**Objective:**

This study aimed to explore the relationship between SS and all-cause mortality in Chinese older adults, as well as the influence of healthy lifestyle factors.

**Methods:**

Three groups of SS were defined through latent class analysis: low, medium, and high. We created a healthy lifestyle index based on smoking, alcohol consumption, physical activity, and diet. Multivariable Cox proportional hazards models, interaction analyses, and mediation analyses were conducted.

**Results:**

The Chinese Longitudinal Healthy Longevity Survey (CLHLS) datasets included participants from 806 cities and counties across 23 provinces in China from 1998 to 2018. In this study, participants were recruited from 4 waves of the CLHLS (2005, 2008, 2011, and 2014). Finally, 19,236 participants were included in this study, of which 6891 (35.8%) had low SS, 11,662 (60.6%) had medium SS, and 683 (3.6%) had high SS. In the fully adjusted model, the hazard ratio (HR) for medium SS was 1.16 (95% CI 1.11‐1.20; *P*<.001), and for high SS, it was 1.28 (95% CI 1.18‐1.40; *P*<.001) compared to the low SS group. For individuals aged ≥80 years, the medium SS group had a 28% (HR 1.28, 95% CI 1.22‐1.34; *P*<.001) increased mortality risk, and the high SS group had a 38% (HR 1.38, 95%CI 1.26‐1.52; *P*<.001) increased risk compared to the low SS group. Approximately 7% of the association between SS and mortality was mediated through the healthy lifestyle. Under different SS, the lower the healthy lifestyle score, the higher the risk of mortality.

**Conclusions:**

SS was an independent predictor of all-cause mortality in Chinese older adults. The healthy lifestyle mediated this effect to some extent. Unhealthy lifestyle behaviors were associated with a higher risk of mortality at all SS levels.

## Introduction

As social progress advances, increasing attention has been given to the impact of chronic social stress (SS) on human health. SS is a complex and multifaceted construct, typically encompassing 3 key areas, such as interpersonal relationships, economic conditions, and the living environment. Interpersonal stress arises from demands and expectations within family, friendships, and work relationships [1]. Economic stress is primarily linked to concerns over income or job stability [2] while environmental stress involves factors, such as housing stress and access to social services [3]. All of these factors can contribute to an individual’s overall level of SS. Clinical and biological studies have demonstrated that long-term exposure to stress can disrupt the normal functioning of biological systems, leading to negative health outcomes, such as cardiovascular disease [4], gastrointestinal disorders [5], and immune dysfunction [6]. In addition, unhealthy lifestyle behaviors, such as poor diet, physical inactivity, and smoking, are well-documented risk factors for a range of chronic diseases and premature mortality [7], and may further compound the adverse effects of SS.

China is undergoing a significant demographic shift, with a rapidly aging population. As such, the health and well-being of older adults have become key areas of focus. While stress affects individuals at all stages of life, its impact on older adults warrants particular attention. Studies have shown that stress may be linked to poorer health in older adults [8-10]. However, more research is needed to understand how SS affects the health of older adults and the impact of lifestyle.

This study aimed to investigate the impact of different levels of SS on mortality among Chinese adults older than 65 years, using data from the Chinese Longitudinal Healthy Longevity Survey (CLHLS). In addition, the study investigated the possible impact of a healthy lifestyle on the relationship between SS and all-cause mortality.

## Methods

### Study Population

The data for this study were drawn from the Chinese Longitudinal Healthy Longevity Survey (CLHLS), a prospective cohort study described in detail in previous publications [11,12]. In brief, the CLHLS was based on a dynamic, multistage stratified sampling design, which includes participants from 806 cities and counties across 23 provinces in China from 1998 to 2018. The survey covers 85% of the Chinese population, with a focus on individuals aged 65 years and older [13]. Data collected include information on demographic characteristics, socioeconomic status, lifestyle factors, and health-related variables. For this analysis, 27,585 participants were recruited from 4 waves of the CLHLS (2005, 2008, 2011, and 2014). During the follow-up surveys, the survival status of the participants was ascertained as surviving, dead, or lost to follow-up. Participants were excluded if they met any of the following criteria: (1) lost to follow-up or dead before (n=5285), (2) age younger than 65 years at baseline (n=201), and (3) missing baseline information (n=2863). After applying these exclusions, the final sample consisted of 19,236 participants.

### Assessment of SS

Existing research primarily examines the impact of SS on health from 3 aspects, namely, interpersonal relationships, economic conditions, and housing conditions [14-16]. Accordingly, our study assessed SS among the older adult population in the CLHLS based on these 3 dimensions. Based on the investigation of relationships with marriage, the health of children, and social support, these questions further transformed into 3 questions, such as “Do you live with your spouse?,” “Are your children still alive?,” and “Do you have someone with whom you can talk, confide in, or seek help when needed?” Economic status was measured by the question, “Is all of the financial support sufficient to pay for daily expenses?” Housing stress was evaluated using the question, “How many people are living with you?” Given the living arrangements typical for older adults in China, those living alone or with more than 5 people were categorized as experiencing housing stress (Table S1 in Multimedia Appendix 1).

### Assessment of Healthy Lifestyle Score

A healthy lifestyle was defined by 4 factors, such as smoking, alcohol consumption, physical activity, and diet. Participants who had never smoked or had quit smoking for at least 30 years were assigned 1 point [17]. Men who consume less than 62 g of alcohol per day or women who consume less than 41 g per day were assigned 1 point [18]. Physical activities included regular exercise (both aerobic and anaerobic), housework, tasks, outdoor activities, gardening, pet care, reading, playing cards or mahjong, watching television or listening to the radio, and attending social events. The frequency of “almost every day” was scored as 2, “occasionally” was scored as 1, and “rarely or never” was scored as 0. A total physical activity score was calculated by summing the points across these categories and then standardized to a range of 0-1. A score below 0.6 was assigned 0 points while a score of 0.6 or above was assigned 1 point (Table S2 in Multimedia Appendix 1). The dietary score was determined using a standardized food frequency questionnaire that includes fresh vegetables, fresh fruits, legumes, meat, eggs, fish and seafood, salty vegetables, tea, and garlic. Similar to physical activity, the score for each food group was based on frequency of consumption, and the total dietary score is calculated accordingly (Table S3 in Multimedia Appendix 1). The final lifestyle score ranged from 0 to 4, with higher scores indicating a healthier lifestyle [19].

### Outcome

Death was confirmed either by official death certificates or through information provided by the participant’s next of kin, local physician, or resident physician. The follow-up period was defined as the time from study enrollment to the date of death or the last follow-up, whichever came first. Loss to follow-up was defined as the inability to contact the participant after at least 3 documented attempts.

### Covariates

Several variables were considered as potential confounding factors. The first set included demographic variables, namely, age (y), sex (male or female), residence (urban or rural), education, and occupation prior to retirement. Considering that the participants included in this study were 65 years old and above, 0 years of education was defined as low, less than 5 years of education was defined as medium, and 5 years of education or more was defined as high. Occupations before retirement were divided into farming, employed, and unemployed. The second set included the history of chronic diseases, such as hypertension (yes or no), diabetes (yes or no), heart disease (yes or no), stroke and cerebrovascular disease (yes or no), and cancer (yes or no). The history of chronic diseases was measured with the following question: “Have you been diagnosed by a doctor with the conditions listed below?”

### Statistical Analysis Methods of the Results

Continuous variables were summarized as means and SDs while categorical variables were presented as counts (N) and percentages (%). Differences between groups were assessed using analysis of variance for continuous variables and the chi-square test for categorical variables. Cox proportional hazard regression models were used to estimate the hazard ratios (HRs) and 95% CI for mortality associated with SS and healthy lifestyle scores. The proportional hazards assumption was tested using Schoenfeld residuals. Model 1 was unadjusted; Model 2 adjusted for age and sex; Model 3 further included education level, occupation, and residence; and Model 4 additionally adjusted for healthy lifestyle factors. Mediation analysis was used to further explore the role of healthy lifestyle in the relationship between SS and all-cause mortality. In addition, the product term of SS and healthy lifestyle score was added to the model to examine their multiplicative interaction. The relative excess risk due to interaction and its corresponding 95% CI were used to assess the interaction on the additive scale. This was calculated using the coefficients and SEs of the product term, SS, and healthy lifestyle score, along with the covariance matrix.

Latent class analysis (LCA) was used to classify participants into distinct SS groups. Convergence was defined as a maximum absolute deviation of 0.000001 between parameter estimates across successive iterations. Iterations terminated when this deviation threshold was reached. Model selection was guided by criteria, including Akaike Information Criterion, Bayesian Information Criterion, likelihood ratio statistic (G^2^), and entropy value. Entropy, ranging from 0 to 1, assesses the accuracy of classification in LCA, with higher values indicating better model classification accuracy. In this study, after convergence failed for the 6-class model, 5 latent class groups were selected for comparison, with optimal model performance observed at 3 latent classes based on the highest entropy values (Tables S4-S6 in Multimedia Appendix 1).

Furthermore, when we analyze the impact of a healthy lifestyle on mortality under different SS conditions, the small number of healthy lifestyle groups in the high SS group may affect the results. Therefore, we combined the medium SS group and the high SS group into the medium-high SS group for analysis. We conducted a subgroup analysis stratified by age, sex, education, residence, and occupation to present the differences. We performed several sensitivity analyses to test the robustness of our results: (1) Excluded deaths that occurred within the first 2 years of follow-up. (2) Excluded the mediating effect of each lifestyle factor on the relationship between SS and all-cause mortality. (3) The results were evaluated again by weighting the lifestyle scores. (4) Due to the low proportion of participants with lifestyle scores of 2 and 3, they were combined into 1 group for analysis.

All analyses were based on R version 4.3.2 (R Foundation for Statistical Computing). All statistical tests were 2-sided, with *P*<.05 considered to be indicative of statistical significance.

### Ethical Considerations

This study was approved by the Ethics Committee of Peking University (IRB00001052 −13074), and informed consent was obtained from all participants. All data were anonymized and deidentified to protect participant privacy. Participants provided written consent for the publication of their anonymized data and quotes in this study. 

## Results

### Baseline Characteristics

**Figure 1.**
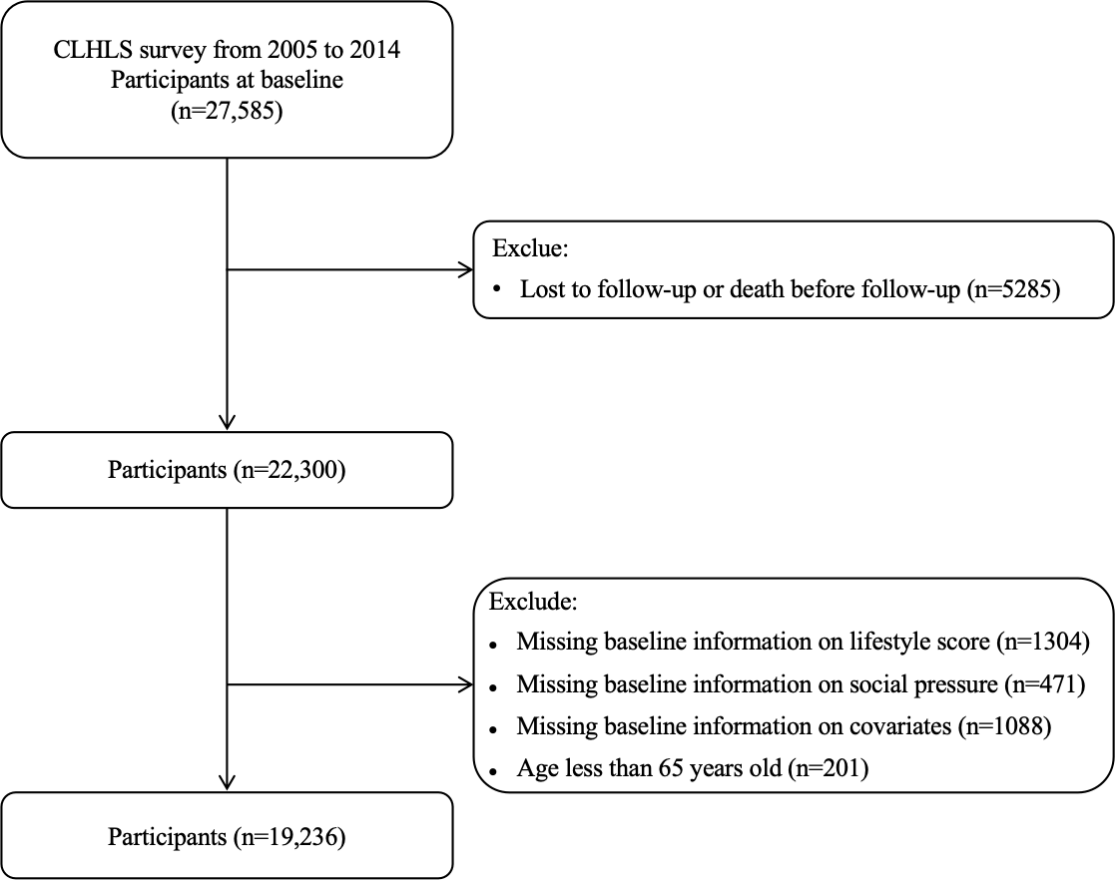
Flowchart of participant selection. CLHLS: Chinese Longitudinal Healthy Longevity Survey.

A total of 19,236 Chinese adults aged 65 years and older were included in this prospective cohort study (Figure 1). Among them, 6891 (35.8%) people had low SS, 11,662 (60.6%) people had medium SS, and 683 (3.6%) people had high SS. The overall mean age of the participants was 88 (SD 12) years. Overall, females, rural residents, medium education, and agriculture make up a larger proportion of the total population. The high SS group exhibited a higher proportion of individuals experiencing several adverse conditions, including the loss of children, lack of social support, living without a spouse, insufficient financial resources to cover daily expenses, and housing stress. Overall, 6827 (35.5%) participants had a healthy lifestyle score of 0, 7017 (36.5%) participants scored 1, 2633 (13.7%) participants scored 2, and 2759 (14.3%) participants scored ≥3. Chronic disease conditions and other details can be seen in Table 1.

**Table 1.** Baseline characteristics of the study participants.

Variables	Overall (N=19,236)	Social stress		
		Low (n=6891)	Medium (n=11,662)	High (n=683)
Age (year), mean (SD)	88 (12)	81 (11)	91 (10)	91 (10)
Sex (female), n (%)	11195 (58.2)	2792 (40.5)	7964 (68.3)	439 (64.3)
Residence (urban), n (%)	2338 (12.2)	908 (13.2)	1368 (11.7)	62 (9.1)
Education^a^, n (%)				
High	3436 (17.9)	1950 (28.3)	1429 (12.3)	57 (8.3)
Medium	12487 (64.9)	3432 (49.8)	8513 (73.0)	542 (79.4)
Low	3313 (17.2)	1509 (21.9)	1720 (14.7)	84 (12.3)
Occupation, n (%)				
Agriculture	13042 (67.8)	4382 (63.6)	8140 (69.8)	520 (76.1)
Unemployment	2560 (13.3)	671 (9.7)	1785 (15.3)	104 (15.2)
Employment	3634 (18.9)	1838 (26.7)	1737 (14.9)	59 (8.6)
Are your children alive? (Yes), n (%)	18519 (96.3)	6810 (98.8)	11232 (96.3)	477 (69.8)
Do you have someone with whom you can talk, confide in, or seek help when needed? (Yes), n (%)	17676 (91.9)	6750 (98.0)	10778 (92.4)	148 (21.7)
Do you live with your spouse? (Yes), n (%)	5637 (29.3)	5616 (81.5)	0 (0.0)	19 (3.1)
Is all of the financial support sufficient to pay for daily expenses? (Yes), n (%)	14914 (77.5)	4381 (63.6)	10532 (90.3)	1 (0.1)
Are you living alone or with ≥5 people? (No), n (%)	11141 (57.9)	5545 (80.5)	5333 (45.7)	263 (38.5)
Healthy lifestyle score^b^, n (%)				
≥3	2759 (14.3)	1190 (17.3)	1540 (13.2)	29 (4.2)
2	2633 (13.7)	1040 (15.1)	1460 (12.5)	133 (19.5)
1	7017 (36.5)	2234 (32.4)	4409 (37.8)	374 (54.8)
0	6827 (35.5)	2427 (35.2)	4253 (36.5)	147 (21.5)
Hypertension (yes), n (%)	4981 (25.9)	2032 (29.5)	2829 (24.3)	120 (17.6)
Diabetes (yes), n (%)	1964 (10.2)	824 (12.0)	1107 (9.5)	33 (4.8)
Heart disease (yes), n (%)	2935 (15.3)	1214 (17.6)	1657 (14.2)	64 (9.4)
Stroke and cerebrovascular disease (yes), n (%)	2158 (11.2)	898 (13.0)	1206 (10.3)	54 (7.9)
Cancer (yes), n (%)	1042 (5.4)	404 (5.9)	618 (5.3)	20 (2.9)
Mortality, n (%)	13999 (72.8)	3986 (57.8)	9425 (80.8)	588 (86.1)

a Education: low (0 y), medium (<5 y), high (≥5 y).

b Healthy lifestyle was defined by 4 factors: smoking, alcohol consumption, physical activity, and diet. Healthy lifestyle score ranges from 0 to 4, with higher scores indicating a healthier lifestyle.

### Association of SS and All-Cause Mortality

In the unadjusted analysis (Model 1), compared with the low SS group, the results showed the HRs of the medium and high SS groups were 2.05 (95% CI 1.98‐2.13) and 2.47 (95% CI 2.26‐2.69). After adjusting for age and sex (Model 2), the HRs of the medium and high SS groups were 1.15 (95% CI 1.11‐1.20) and 1.37 (95% CI 1.26‐1.50). In Model 3, which further adjusted for local education, occupation, and healthy lifestyle score based on Model 2, the HRs of the medium and high SS groups were 1.15 (95% CI 1.11‐1.20) and 1.28 (95% CI 1.17‐1.39). In the fully adjusted Model 4, the mortality risk for medium SS was 1.16 (95% CI 1.11‐1.20), and for high SS, it was 1.28 (95% CI 1.18‐1.40) compared to the low SS groups (Table 2).

**Table 2.** Association of social stress and all-cause mortality.

Social stress	Model 1^a^ HR^b^ (95% CI)	Model 2^c^ HR (95% CI)	Model 3^d^ HR (95% CI)	Model 4^e^ HR (95% CI)	*P* value
Low^f^	1 (Reference)	1 (Reference)	1 (Reference)	1 (Reference)	—^g^
Medium	2.05 (1.98-2.13)	1.15 (1.11-1.20)	1.15 (1.11-1.20)	1.16 (1.11-1.20)	<.001
High	2.47 (2.26-2.69)	1.37 (1.26-1.50)	1.28 (1.17-1.39)	1.28 (1.18-1.40)	<.001

a Model 1: unadjusted.

b Hazard ratio

c Model 2: adjusted for age and sex.

d Model 3: adjusted for residence, education, occupation, and healthy lifestyle score based on Model 2.

e Model 4: adjusted for hypertension, diabetes, heart disease, cancer, stroke, and cerebrovascular disease based on Model 3.

f The group is set as a reference group.

g Not applicable.

### Impact of a Healthy Lifestyle on All-Cause Mortality Across Different SS Groups

**Figure 2.**
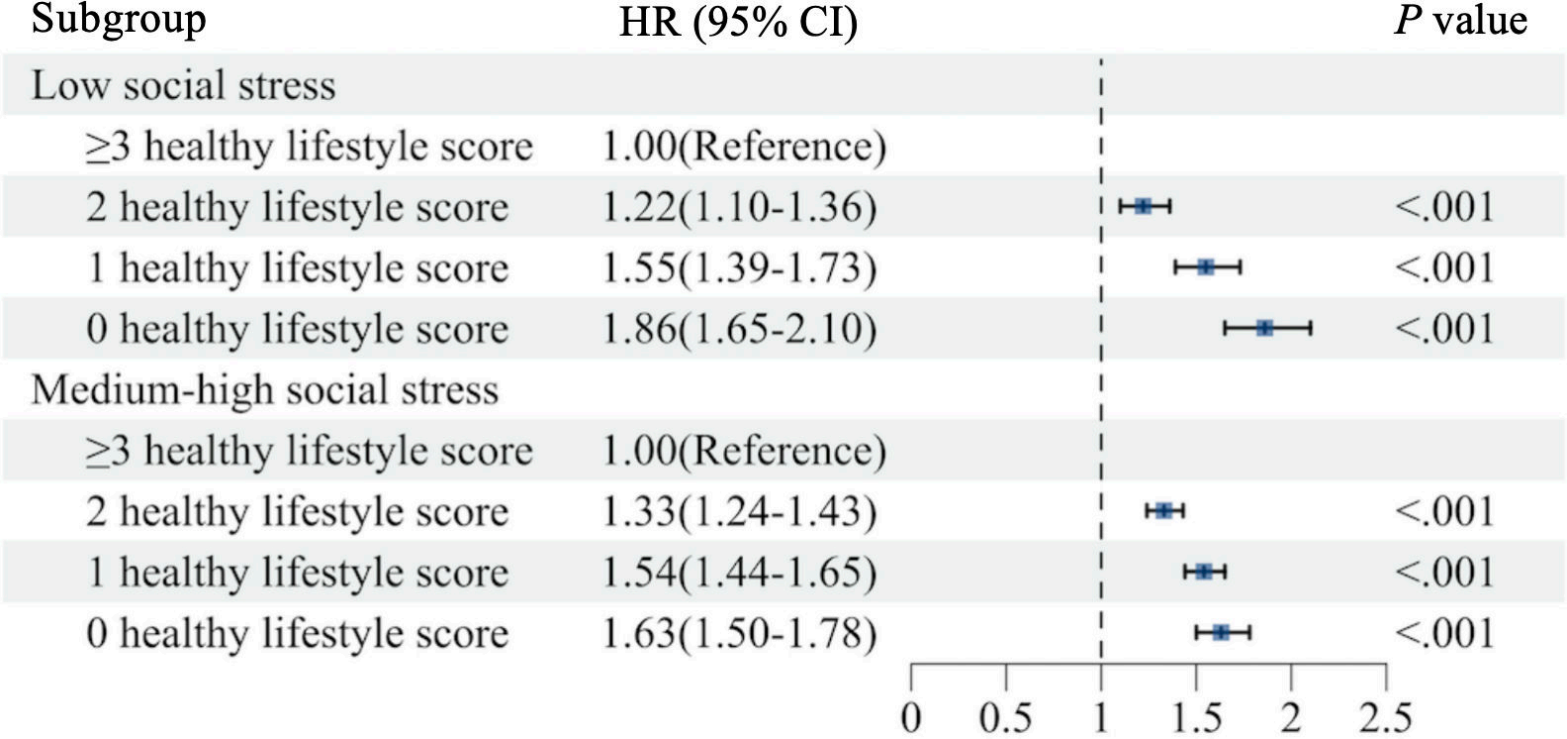
Impact of a healthy lifestyle on all-cause mortality across different social stress groups. All covariates were adjusted. HR: hazard ratio.

The lower the healthy lifestyle score, the higher the risk of death compared with a healthy lifestyle score ≥3 (Table S7 in Multimedia Appendix 1). For the low SS group, individuals with a higher healthy lifestyle score had a lower mortality risk. Specifically, compared to those with a healthy lifestyle score of 3 or more, individuals with a score of 2 had a 22% increased risk of mortality (HR 1.22, 95% CI: 1.10‐1.36), those with the score of 1 had a 55% increased risk (HR 1.55, 95% CI 1.39‐1.73), and those with the score of 0 had an 86% increased risk (HR 1.86, 95% CI 1.65‐2.10). Further combining the medium and high SS groups, the results found that individuals with a score of 2 had a 33% higher mortality risk (HR 1.33, 95% CI 1.24‐1.43) compared to those with a score of 3 or more. The score of 1 was associated with a 54% higher risk (HR 1.54, 95% CI 1.44‐1.65), and the score of 0 was associated with a 63% higher risk (HR 1.63, 95% CI 1.50‐1.78) (Figure 2).

### Mediation and Interaction Analysis of a Healthy Lifestyle on Associations of SS With All-Cause Mortality

**Figure 3.**
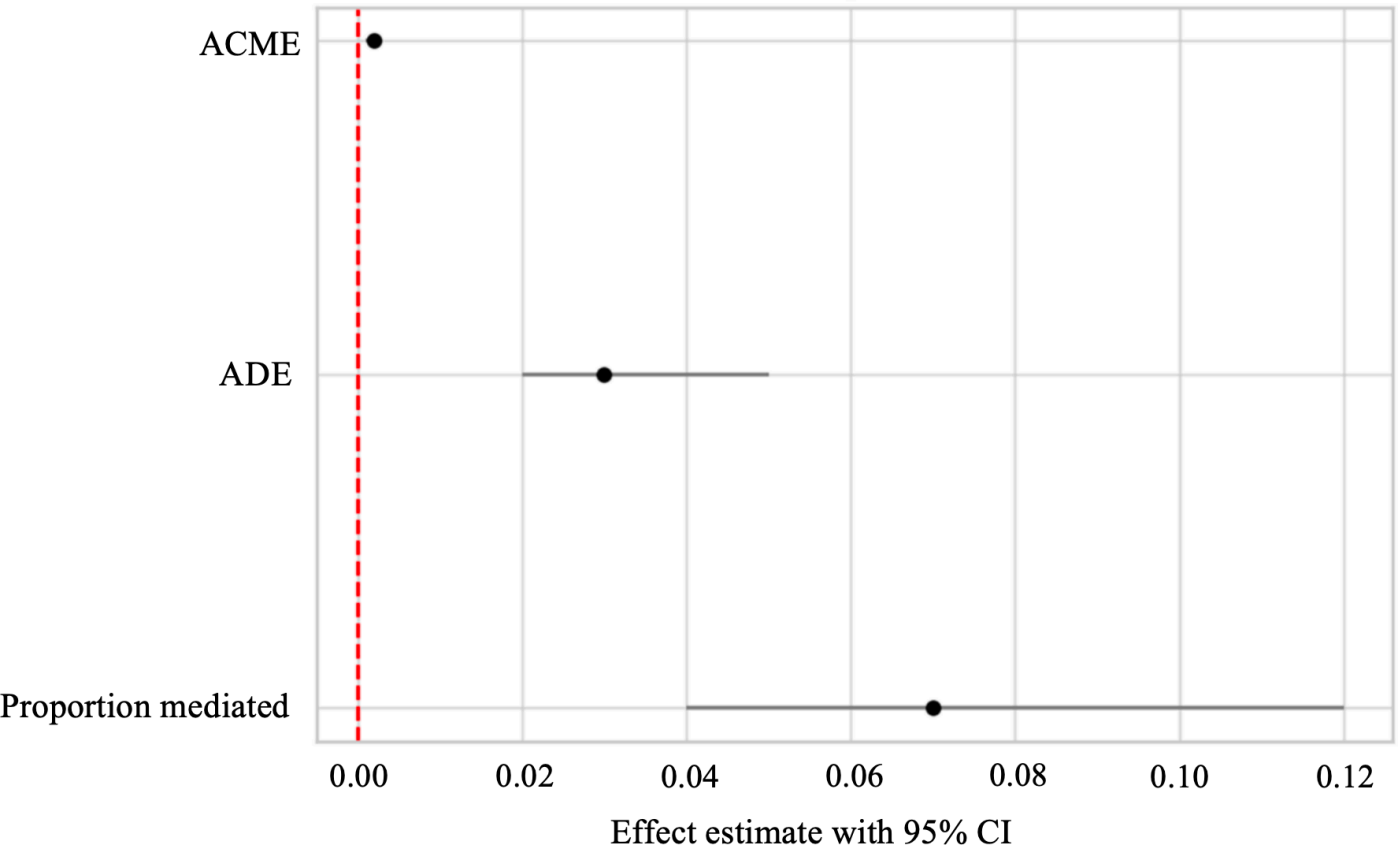
The mediating effect of a healthy lifestyle in the relationship between social stress and all-cause mortality. ACME: average causal mediation effects; ADE: average direct effects.

The proportion of the total effect of SS on all-cause mortality that is mediated through a healthy lifestyle was 0.07 (95% CI 0.04-0.12), with a *P* value <.001. This suggests that approximately 7% of the association between SS and mortality is explained by the mediation effect of a healthy lifestyle. The average direct effect of SS on all-cause mortality, independent of the mediation through a healthy lifestyle, was 0.03 (95% CI 0.02-0.05), with a *P* value <.001. The average causal mediation effect was 0.002 (95% CI 0.001-0.002), with a *P* value <.001. These findings demonstrate that a healthy lifestyle partially mediates the relationship between SS and all-cause mortality (Figure 3).

After multiplicative interaction, the interaction term exponentiated coefficient (exp [coef]) is 0.941, and the *P* value was less than .001, indicating that there was a significant multiplicative interaction between social pressure and a healthy lifestyle. The additive interaction results showed that the relative excess risk due to interaction was measured to be 0.127 (95% CI 0.013‐0.240), indicating the existence of a positive additive interaction. The attributable proportion due to interaction was found to be 0.071 (95% CI 0.017‐0.124), indicating that 7.1% of the combined mortality risk caused by social pressure and unhealthy lifestyle can be attributed to the synergistic effect of both factors. The Synergy Index was calculated to be 1.189 (95% CI 1.060‐1.334), indicating that when SS coexists with unhealthy lifestyles, the risk of death is 1.189 times higher than the sum of their individual effects.

### Stratified and Sensitivity Analyses

We analyzed the effects of SS and a healthy lifestyle on the oldest- and nonoldest-old. The results showed that there was a significant association between SS and mortality risk in both the ≥80 years and <80 years age groups (Table S8 in Multimedia Appendix 1). In the older adults and nonolder adults groups, regardless of stress level, the mortality risk (HR) increased significantly as the healthy lifestyle score decreased (from ≥3 points to 0 points) (Figure S1 and Table S9 in Multimedia Appendix 1). In the older adults and nonolder adults groups, healthy lifestyle partially mediated the relationship between SS and mortality risk (Table S10 in Multimedia Appendix 1). The associations of SS, healthy lifestyle, and all-cause mortality were generally similar across sex (Table S11 in Multimedia Appendix 1), residence (Table S12 in Multimedia Appendix 1), occupation (Table S13 in Multimedia Appendix 1), and education (Table S14 in Multimedia Appendix 1). The associations of SS, healthy lifestyle, and all-cause mortality did not change appreciably when we excluded participants who died within 1 or 2 years of follow-up (Table S15 in Multimedia Appendix 1). The mediating effects of different components of a healthy lifestyle (smoking, alcohol consumption, physical activity, and diet scores) on the relationship between SS and mortality were further analyzed (Table S16 in Multimedia Appendix 1). Association of SS with all-cause mortality after adjustment for weighted lifestyle score and other covariates (Table S17 in Multimedia Appendix 1). Since the population with lifestyle score=2 and lifestyle score=3 each accounted for 1/3 of the other population (lifestyle score=0 or 1), we combined the population with score 2 and score 3 for sensitivity analysis, and the results remained robust (Table S18 in Multimedia Appendix 1).

## Discussion

### Principal Findings

In this large prospective cohort study, we investigated the impact of SS and a healthy lifestyle on mortality among older adults in China. Our findings showed that both medium and high levels of SS were associated with increased mortality risks, with HRs of 1.16 and 1.28, respectively, compared to the low SS group after full adjustment for covariates. For individuals aged <80 years and ≥80 years, the HRs of the high SS group were 2.09 and 1.38. SS has a greater impact on the mortality of older adults younger than 80 years. These results underscore the significant impact of SS on health outcomes in older adults. Approximately 7% of the association between SS and mortality was mediated through the healthy lifestyle. Under different SS, the lower the healthy lifestyle score, the higher the risk of mortality. These results provide valuable insights into the complex interplay between SS and health behaviors in shaping mortality risks among older adults in China.

Chronic SS has been shown to induce a range of physiological and behavioral changes that contribute to the development or progression of various health conditions [20]. Previous studies have demonstrated that prolonged exposure to stress elevates plasma cortisol levels, which may lead to memory impairment [21]. Mild stress could help improve cognitive function, but if the intensity of stress exceeded a predetermined threshold (which is different for each person), it could lead to cognitive impairment [22]. Stress could affect the function of the immune system by regulating the processes of the central nervous system and the neuroendocrine system [23]. In addition, stress could affect the occurrence of cardiovascular disease, gastrointestinal disease, endocrine disease, and other diseases [24-26]. A study from China explored the relationship between SS and health inequality and confirmed that SS has a greater impact on people with low economic status [14]. However, most existing studies focus on the impact of SS on psychological or psychiatric diseases. In addition, there is currently a lack of research on the impact of SS on the survival of Chinese older adults and the role of lifestyle in this association. With the aging trend in China, it is necessary to explore the relationship between SS and the health of older adults [27,28]. In our study, we found that compared with low SS, moderate and high SS increased the risk of death in Chinese people aged 65 years and more than 65 years to varying degrees, and lifestyle played a partial mediating role. This study provides causal support for the relationship between SS and the health of older adults through prospective analysis.

Although stress and its effects occur at all stages of an individual’s life, with the rapid development of China’s aging population, the social pressure faced by older adults has increased, mainly including pressure from interpersonal relationships, the economy, and the environment [29,30]. These components are interrelated and should be examined collectively in relation to health outcomes in older adults. Our study divided these 3 variables into 3 levels, such as low, medium, and high, based on LCA analysis. The results showed that among adults aged 65 years and older, compared with low SS, medium SS increased the risk of death by 16%, and high SS increased the risk of death by 28%. Approximately 7% of the association between SS and mortality is explained by the mediation effect of a healthy lifestyle. This indicates that SS directly influences mortality risk among older adults, and although a healthy lifestyle partially mediates this relationship, the effect size is small. We speculated that there may be several reasons. First, for older adults, the impact of SS may have already had a cumulative effect on their physical health earlier in life, and adopting a healthy lifestyle may not be sufficient to fully offset this long-term negative impact. Second, with age, declining physical functioning, accumulated chronic diseases, and a weakened immune system may reduce older adults’ ability to benefit from a healthy lifestyle. Third, social pressure is a complex stressor that may affect the health of older adults’ pathways, such as mental well-being, immune function, quality of life, and other mechanisms. Older adults may face higher levels of loneliness and reduced social support, and a healthy lifestyle may not sufficiently mitigate the physiological and psychological effects of these pressures.

Furthermore, participants aged ≥80 years were analyzed separately from younger older adults. We found that SS had a greater impact on mortality risk among older adults aged less than 80 years. Compared with the low SS group, mortality risk increased by 35% and 109% in the medium and high SS groups, respectively. Among adults aged 80 years and older than 80 years, the risk of death increased by 28% and 38% in the medium and high SS groups, respectively, compared to the low SS group. This suggests that SS may pose a greater threat to the survival of younger-old adults. Several factors may help explain this phenomenon. First, younger-old adults may be in a critical stage of lifestyle change, such as facing adaptation after retirement, changes in social roles, etc. Stress may accelerate the occurrence or aggravation of cardiovascular diseases, diabetes, and other chronic diseases, potentially leading to premature death [31]. Second, younger-older adults may not fully cope with SS, resulting in a surge in emotions, such as anxiety and depression [32]. Third, younger-older adults may experience greater economic pressure, including ongoing family expenses and housing challenges [33]. Fourth, in response to a rapidly changing social environment, younger-old adults may adopt unhealthy coping strategies, such as smoking or alcohol consumption, which can exacerbate health risks [34]. Therefore, our findings suggest that age-specific interventions may be necessary to reduce the impact of SS on the health and survival of older adults. This study investigated the interaction between SS and healthy lifestyle on all-cause mortality, using both multiplicative and additive interaction models. The findings revealed a statistically significant interaction effect, suggesting that the relationship between SS and mortality is modified by a healthy lifestyle. The additive interaction highlights the importance of addressing both SS and unhealthy lifestyles simultaneously. Interventions targeting stress reduction alongside lifestyle promotion could yield greater mortality benefits than addressing either factor in isolation.

Studies have shown that SS affects health through a variety of biological pathways. For example, long-term SS may increase cortisol levels in the body, which may lead to decreased immune function and enhanced inflammatory response, thereby increasing the risk of illness or death [35]. Our study suggests that reducing SS among older adults may help improve their quality of life. Moreover, our study found that social pressure had a greater impact on mortality risk among males, as well as among older adults who lived in urban areas, had never been employed, and had received more than 5 years of education. These findings offer valuable insights for future research on SS among older adults.

Our study has several major strengths, including a large sample size and a prospective cohort study. To our knowledge, no previous study has provided a detailed analysis of SS and mortality among the older adult population in China. Nevertheless, our study also has some limitations. First, data related to SS and data related to healthy lifestyles were based on self-reports, which, despite strict collection criteria, are still prone to measurement errors. Second, cause-of-death data were not comprehensively recorded, limiting our ability to assess the impact of SS on cause-specific mortality among older adults. Third, residual confounding could not be fully eliminated due to limited covariates, such as the absence of genetic data and information on other health conditions. Fourth, this study only focuses on the social pressure composed of interpersonal relationships, economic status, and living environment and may not have considered other related factors. Fifth, although our lifestyle score included key behavioral factors, such as smoking, diet, physical activity, and alcohol consumption, other potentially important components, such as sleep duration and quality, perceived stress, and sedentary behavior, were not included due to data limitations. Sixth, we did not consider the potential changes in lifestyle factors during this period, which may affect the observed results. Therefore, more relevant studies are needed to provide evidence for the present findings.

### Conclusions

The results showed that SS was an important risk factor for all-cause mortality in the Chinese older adults. This association remained strong even after adjusting for various sociodemographic and health-related factors, highlighting the importance of SS as a determinant of mortality risk in the Chinese older adults. A healthy lifestyle only partially mediated the relationship between SS and survival among older adults. However, an unhealthy lifestyle was associated with an increased risk of mortality, regardless of SS level.

## Supplementary material

10.2196/75942 Multimedia Appendix 1 Additional data supporting the primary analysis.

## Abbreviations

CLHLS: Chinese Longitudinal Healthy Longevity Survey
HR: hazard ratio
LCA: latent class analysis
SS: social stress
