# Supplementary material for: The Impact of Social Stress and Healthy Lifestyle on the Mortality of Chinese Older Adults: Prospective Cohort Study
Source: JMIR Aging. 2025 Aug 12;8:e75942. doi: 10.2196/75942 (PMC12342692; doi:10.2196/75942)
Supplement: Multimedia Appendix 1 [file aging-v8-e75942-s001.docx]

**Supplementary materials**

[Table S1. Definition of social stress 2](#_Toc1693021584)

[Table S2. Definition of each component of a healthy lifestyle score 3](#_Toc455439538)

[Table S3. Definition of each component of a healthy diet score 4](#_Toc940597258)

[Table S4 . Assessment of social stress using latent class analysis 5](#_Toc990989639)

[Table S5. Sample size of three latent classes by social stress 6](#_Toc1827180188)

[Table S6. Practical definitions of low, medium and high social stress in the three-latent-class 7](#_Toc401267616)

[Table S7. The relationship between healthy lifestyle and all-cause mortality 9](#_Toc1006170532)

[Table S8. Association of social stress and all-cause mortality by age group 10](#_Toc1421894846)

[Table S9. Impact of healthy lifestyle on all-cause mortality across different social stress group by age group 11](#_Toc588652906)

[Table S10. The mediating effect of healthy lifestyle on the relationship between social stress and all-cause mortality by age group 12](#_Toc32229413)

[Table S11. Sex stratified analysis of the impact of social stress and healthy lifestyle on mortality 13](#_Toc513865247)

[Table S12. Residence stratified analysis of the impact of social stress and healthy lifestyle on mortality 14](#_Toc1501461742)

[Table S13. Occupation stratified analysis of the impact of social stress and healthy lifestyle on mortality 15](#_Toc2134645544)

[Table S14. Education stratified analysis of the impact of social stress and healthy lifestyle on mortality 16](#_Toc1125851226)

[Table S15.Association of social stress, healthy lifestyle and mortality 17](#_Toc703141665)

[Table S16. The mediating role of lifestyle factors (smoking, alcohol consumption, physical activity, and diet score) in the relationship between social stress and all-cause mortality 18](#_Toc99454214)

[Table S17. Association of SS with all-cause mortality after adjustment for weighted lifestyle score and other covariates 19](#_Toc784697332)

[Table S18. Association of social stress, healthy lifestyle (reclassification) and mortality 20](#_Toc710982697)

[Supplementary Figure 1. Impact of healthy lifestyle on all-cause mortality across different social stress group by age group 21](#_Toc887176571)

## Table S1. Definition of social stress

| **Social stress** | **Category** | **Score** |
| --- | --- | --- |
| Someone you can talk to, confide in, or seek help when needed | spouse/son/daughter/daughter in law/son in law/grandchildren and their spouses/other relatives/friends/neighbors/social occupationers/housekeeper | 1 |
|  | nobody | 0 |
| Living with spouse | currently married and living with spouse | 1 |
|  | separated/divorced/widowed/never married | 0 |
| Economy pressure | yes | 0 |
|  | no | 1 |
| Residential environment pressure | 2-4 people | 1 |
|  | 1 person and 5 or more people | 0 |
| Are your children still alive | yes | 1 |
|  | no | 0 |

## Table S2. Definition of each component of a healthy lifestyle score

| **Physical activity** | **Category** | **Score** |
| --- | --- | --- |
| Regular  exercise | yes | 2 |
|  | no | 0 |
| Housework  tasks | almost everyday/not every day, but at least once a week | 2 |
|  | not every week, but at least once a month | 1 |
|  | not every month, but sometimes/never | 0 |
| Personal  outdoor activities | almost everyday/not every day, but at least once a week | 2 |
|  | not every week, but at least once a month | 1 |
|  | not every month, but sometimes/never | 0 |
| Gardening | almost everyday/not every day, but at least once a week | 2 |
|  | not every week, but at least once a month | 1 |
|  | not every month, but sometimes/never | 0 |
| Rearing domestic animals/pets | almost everyday/not every day, but at least once a week | 2 |
|  | not every week, but at least once a month | 1 |
|  | not every month, but sometimes/never | 0 |
| Reading | almost everyday/not every day, but at least once a week | 2 |
|  | not every week, but at least once a month | 1 |
|  | not every month, but sometimes/never | 0 |
| Playing  cards/mahjong | almost everyday/not every day, but at least once a week | 2 |
|  | not every week, but at least once a month | 1 |
|  | not every month, but sometimes/never | 0 |
| Watching  tv/ listening to the radio | almost everyday/not every day, but at least once a week | 2 |
|  | not every week, but at least once a month | 1 |
|  | not every month, but sometimes/never | 0 |
| Attending  social activities | almost everyday/not every day, but at least once a week | 2 |
|  | not every week, but at least once a month | 1 |
|  | not every month, but sometimes/never | 0 |

## Table S3. Definition of each component of a healthy diet score

| **Diet intake** | **Category** | **Score** |
| --- | --- | --- |
| Fresh  vegetables | everyday or almost everyday/quite often | 2 |
|  | occasionally | 1 |
|  | rarely or never | 0 |
| Fresh  fruit | everyday or almost everyday/quite often | 2 |
|  | occasionally | 1 |
|  | rarely or never | 0 |
| Legumes | almost everyday/ not every day, but at least once a week | 2 |
|  | not every week, but at least once a month | 1 |
|  | not every month, but occasionally/rarely or never | 0 |
| Meat | almost everyday/ not every day, but at least once a week | 2 |
|  | not every week, but at least once a month | 1 |
|  | not every month, but occasionally/rarely or never | 0 |
| Egg | almost everyday/ not every day, but at least once a week | 2 |
|  | not every week, but at least once a month | 1 |
|  | not every month, but occasionally/rarely or never | 0 |
| Fish and seafood | almost everyday/ not every day, but at least once a week | 2 |
|  | not every week, but at least once a month | 1 |
|  | not every month, but occasionally/rarely or never | 0 |
| Salty  vegetables | almost everyday/ not every day, but at least once a week | 0 |
|  | not every week, but at least once a month | 1 |
|  | not every month, but occasionally/rarely or never | 2 |
| Tea | almost everyday/ not every day, but at least once a week | 2 |
|  | not every week, but at least once a month | 1 |
|  | not every month, but occasionally/rarely or never | 0 |
| Garlic | almost everyday/ not every day, but at least once a week | 2 |
|  | not every week, but at least once a month | 1 |
|  | not every month, but occasionally/rarely or never | 0 |

## Table S4 . Assessment of social stress using latent class analysis

| **Number of Class** | **Log-likelihood** | **residual degrees of freedom** | **G^2a^** | **AIC^b^** | **BIC^c^** | **Entropy** |
| --- | --- | --- | --- | --- | --- | --- |
| 2 | -42622.31 | 20 | 248.461 | 85266.62 | 85353.13 | 0.428 |
| 3 | -45831.66 | 14 | 38.60777 | 85068.77 | 85202.47 | 0.526 |
| 4 | -42511.38 | 8 | 26.59742 | 85068.76 | 85249.64 | 0.499 |
| 5 | -42501.43 | 2 | 6.696852 | 85060.86 | 85288.93 | 0.498 |

**^a^**:Likelihood Ratio Chi-Square Statistic

**^b^**:Akaike Information Criterion

**^c^**:Bayesian Information Criterion

## Table S5. Sample size of three latent classes by social stress

| **Variable** | **Class** | **N(%)** |
| --- | --- | --- |
| **Social stress** | Low social stress | 6891(35.8%) |
|  | Medium social stress | 11662(60.6%) |
|  | High social stress | 683(3.6%) |

## Table S6. Practical definitions of low, medium and high social stress in the three-latent-class

| Are you living alone or≥5 people | Are your children alive | Do you have someone with whom you can talk, confide in, or seek help when needed | Do you live with your spouse | Is all of the financial support sufficient to pay for daily expenses | Frequence |
| --- | --- | --- | --- | --- | --- |
| No | Yes | No | Yes | No | 0.21%a |
| Yes | Yes | No | Yes | No | 0.10%^c^ |
| No | No | No | Yes | No | 0.01%^c^ |
| Yes | No | No | Yes | No | 0.00%^b^ |
| No | Yes | Yes | Yes | No | 4.54%a |
| Yes | Yes | Yes | Yes | No | 1.52%a |
| No | No | Yes | Yes | No | 0.12%a |
| Yes | No | Yes | Yes | No | 0.03%a |
| No | Yes | No | No | No | 1.02%^c^ |
| Yes | Yes | No | No | No | 1.36%^c^ |
| No | No | No | No | No | 0.07%^c^ |
| Yes | No | No | No | No | 0.22%^c^ |
| No | Yes | Yes | No | No | 6.63%a |
| Yes | Yes | Yes | No | No | 5.87%^b^ |
| No | No | Yes | No | No | 0.28%^c^ |
| Yes | No | Yes | No | No | 0.49%^c^ |
| No | Yes | No | Yes | Yes | 0.42%a |
| Yes | Yes | No | Yes | Yes | 0.09%a |
| No | No | No | Yes | Yes | 0.02%a |
| Yes | No | No | Yes | Yes | 0.01%^c^ |
| No | Yes | Yes | Yes | Yes | 16.69%a |
| Yes | Yes | Yes | Yes | Yes | 5.31%a |
| No | No | Yes | Yes | Yes | 0.20%a |
| Yes | No | Yes | Yes | Yes | 0.05%a |
| No | Yes | No | No | Yes | 1.99%^b^ |
| Yes | Yes | No | No | Yes | 2.31%^b^ |
| No | No | No | No | Yes | 0.13%^b^ |
| Yes | No | No | No | Yes | 0.17%^b^ |
| No | Yes | Yes | No | Yes | 24.81%^b^ |
| Yes | Yes | Yes | No | Yes | 23.41%^b^ |
| No | No | Yes | No | Yes | 0.80%^b^ |
| Yes | No | Yes | No | Yes | 1.14%^b^ |

^a^Yellow: low social stress, the proportion of people who answered “NO” to these questions was low: Are your children alive? Do you have someone with whom you can talk, confide in, or seek help when needed? Do you live with your spouse? Is all of the financial support sufficient to pay for daily expenses?

^b^Black: medium social stress, the proportion of people who answered “NO” to these questions was relative high: Are your children alive? Do you have someone with whom you can talk, confide in, or seek help when needed? Do you live with your spouse?

^c^Red: high social stress, the proportion of people who answered “NO” to these questions was high: Are your children alive? Do you have someone with whom you can talk, confide in, or seek help when needed? Do you live with your spouse? Is all of the financial support sufficient to pay for daily expenses?

## Table S7. The relationship between healthy lifestyle and all-cause mortality

| **Variable** | **HR(95%CI)^a^** | **P-value** |
| --- | --- | --- |
| Healthy lifestyle score |  |  |
| ≥3 | 1 (Reference) | - |
| 2 | 1.30(1.22, 1.37) | <.001 |
| 1 | 1.53(1.45, 1.61) | <.001 |
| 0 | 1.70(1.58, 1.82) | <.001 |
| Healthy lifestyle score |  |  |
| One-point decrease in the healthy lifestyle score | 1.19(1.16, 1.21) | <.001 |

All covariates were adjusted.

**^a^**:Hazard Ratio

## Table S8. Association of social stress and all-cause mortality by age group

| **Social stress** | **Model 1^a^** | | **Model 2^b^** | | **Model 3^c^** | | **Model 4^d^** | |
| --- | --- | --- | --- | --- | --- | --- | --- | --- |
| **≥80 years** | **HR(95%CI)^e^** | *P*-value | **HR(95%CI)^e^** | *P*-value | **HR(95%CI)^e^** | *P*-value | **HR(95%CI)^e^** | *P*-value |
| Low | 1 (Reference) |  | 1 (Reference) |  | 1 (Reference) |  | 1 (Reference) |  |
| Medium | 1.27(1.22,1.33) | <.001 | 1.28(1.22, 1.34) | <0.001 | 1.27(1.22, 1.33) | <.001 | 1.28(1.22, 1.34) | <0.001 |
| High | 1.50(1.36,1.64) | <.001 | 1.50(1.37, 1.65) | <0.001 | 1.37(1.24, 1.51) | <0.001 | 1.38(1.26, 1.52) | <0.001 |
| **<80 years** |  |  |  |  |  |  |  |  |
| Low | 1 (Reference) |  | 1 (Reference) |  | 1 (Reference) |  | 1 (Reference) |  |
| Medium | 1.28(1.17,1.41) | <.001 | 1.40(1.27, 1.54) | <0.001 | 1.32(1.20, 1.46) | <.001 | 1.35(1.23,1.48) | <0.001 |
| High | 2.42(1.90,3.07) | <.001 | 2.45(1.93, 3,12) | <0.001 | 2.02(1.58, 2.57) | <.001 | 2.09(1.64,2.66) | <0.001 |

**^a^**Model 1.Unadjusted.

**^b^**Model 2.Adjusted for age and sex.

**^c^**Model 3.Adjusted for local, education, work and healthy lifestyle score based on model 2.

**^d^**Model 4.Adjusted for hypertension, diabetes, heart disease, cancer, stroke and cerebrovascular disease based on model 3.

**^e^**:Hazard Ratio

|  | **≥80 years** | | **<80 years** | |
| --- | --- | --- | --- | --- |
| **Subgroup** | **HR(95%CI)^a^** | ***P-*value** | **HR(95%CI)^a^** | ***P-*value** |
| Low stress |  |  |  |  |
| ≥3 healthy lifestyle score | 1 (Reference) |  | 1 (Reference) |  |
| 2 healthy lifestyle score | 1.49(1.38, 1.50) | .002 | 1.32(1.11, 1.57) | .002 |
| 1 healthy lifestyle score | 1.70(1.58, 1.84) | <.001 | 1.74(1.45, 2.10) | <.001 |
| 0 healthy lifestyle score | 1.81(1.66, 1.99) | <.001 | 2.16(1.74, 2.67) | .002 |
| Medium-high stress |  |  |  |  |
| ≥3 healthy lifestyle score | 1 (Reference) |  | 1 (Reference) |  |
| 2 healthy lifestyle score | 1.49(1.38, 1.61) | <.001 | 1.23(1.00, 1.52) | .005 |
| 1 healthy lifestyle score | 1.71(1.59, 1.84) | <.001 | 1.56(1.25, 1.94) | <.001 |
| 0 healthy lifestyle score | 1.80(1.65, 1.97) | <.001 | 1.23(1.00, 1.52) | .050 |

## Table S9. Impact of healthy lifestyle on all-cause mortality across different social stress group by age group

All covariates were adjusted.

**^a^:**Hazard Ratio

## Table S10. The mediating effect of healthy lifestyle on the relationship between social stress and all-cause mortality by age group

| **Mediation analysis** | **P for Prop. Mediated** | **Prop. Mediated** | **P for ADE^a^** | **ADE** | **P for ACME^b^** | **ACME** |
| --- | --- | --- | --- | --- | --- | --- |
| ≥80 years | <.001 | 0.14  (0.07, 0.36) | <.001 | 0.04  (0.01, 0.06) | <.001 | 0.01  (0.003, 0.01) |
| <80 years | .004 | 0.05  (0.02, 0.10) | <.001 | 0.04  (0.02, 0.05) | .004 | 0.002  (0.006, 0.001) |

**^a^**:Average Direct Effect

**^b^**:Average Causal Mediation Effect

## Table S11. Sex stratified analysis of the impact of social stress and healthy lifestyle on mortality

| **Variable** | **Male** | | **Female** | |
| --- | --- | --- | --- | --- |
|  | **HR(95%CI)^a^** | ***P-*value** | **HR(95%CI)^a^** | ***P-*value** |
| Social stress |  |  |  |  |
| Low | 1 (Reference) | - | 1 (Reference) | - |
| Medium | 1.17(1.10, 1.24) | <.001 | 1.14(1.08, 1.20) | <.001 |
| High | 1.39(1.20, 1.61) | <.001 | 1.17(1.05, 1.31) | <.001 |
| Healthy lifestyle score |  |  |  |  |
| ≥3 | 1 (Reference) | - | 1 (Reference) | - |
| 2 | 1.38(1.25, 1.53) | <.001 | 1.25(1.17, 1.35) | <.001 |
| 1 | 1.60(1.45, 1.76) | <.001 | 1.50(1.40, 1.62) | <.001 |
| 0 | 1.79(1.62, 1.99) | <.001 | 1.63(1.48, 1.81) | <.001 |

**^a^:**Hazard Ratio

## Table S12. Residence stratified analysis of the impact of social stress and healthy lifestyle on mortality

| **Variable** | **Rural** | | **Urban** | |
| --- | --- | --- | --- | --- |
|  | **HR(95%CI)^a^** | ***P-*value** | **HR(95%CI)^a^** | ***P-*value** |
| Social stress |  |  |  |  |
| Low | 1 (Reference) | - | 1 (Reference) | - |
| Medium | 1.15(1.11, 1.21) | <.001 | 1.16(1.03, 1.31) | .016 |
| High | 1.29(1.17, 1.41) | <.001 | 1.30(0.96, 1.76) | .087 |
| Healthy lifestyle score |  |  |  |  |
| ≥3 | 1 (Reference) | - | 1 (Reference) | - |
| 2 | 1.31(1.23, 1.40) | <.001 | 1.20(1.03, 1.38) | .016 |
| 1 | 1.53(1.44, 1.63) | <.001 | 1.58(1.36, 1.83) | <.001 |
| 0 | 1.70(1.57, 1.83) | <.001 | 1.76(1.44, 2.15) | <.001 |

**^a^:**Hazard Ratio

## Table S13. Occupation stratified analysis of the impact of social stress and healthy lifestyle on mortality

| **Variable** | **Agriculture** | | **Unemployment** | | **Employment** | |
| --- | --- | --- | --- | --- | --- | --- |
|  | **HR(95%CI)^a^** | ***P-*value** | **HR(95%CI)^a^** | ***P-*value** | **HR(95%CI)^a^** | ***P-*value** |
| Social stress |  |  |  |  |  |  |
| Low | 1 (Reference) | - | 1 (Reference) | - | 1 (Reference) | - |
| Medium | 1.14(1.09, 1.20) | <.001 | 1.22(1.09, 1.37) | <.001 | 1.14(1.04, 1.26) | .19 |
| High | 1.28(1.16, 1.42) | <.001 | 1.35(1.07, 1.70) | .010 | 1.23(0.90, 1.66) | .007 |
| Healthy lifestyle score |  |  |  |  |  |  |
| ≥3 | 1 (Reference) | - | 1 (Reference) | - | 1 (Reference) | - |
| 2 | 1.24(1.15, 1.33) | <.001 | 1.31(1.13, 1.52) | <.001 | 1.43(1.26, 1.62) | <.001 |
| 1 | 1.44(1.34, 1.55) | <.001 | 1.65(1.42, 1.91) | <.001 | 1.73(1.52, 1.97) | <.001 |
| 0 | 1.58(1.45, 1.72) | <.001 | 1.77(1.47, 2.13) | <.001 | 2.03(1.74, 2.37) | <.001 |

**^a^:**Hazard Ratio

## Table S14. Education stratified analysis of the impact of social stress and healthy lifestyle on mortality

| **Variable** | **High education^a^** | | **Medium education^b^** | | **Low education^c^** | |
| --- | --- | --- | --- | --- | --- | --- |
|  | **HR(95%CI)^d^** | ***P-*value** | **HR(95%CI)^d^** | ***P-*value** | **HR(95%CI)^d^** | ***P-*value** |
| social stress |  |  |  |  |  |  |
| Low | 1 (Reference) | - | 1 (Reference) | - | 1 (Reference) | - |
| Medium | 1.25(1.12, 1.38) | <.001 | 1.11(1.01, 1.22) | .001 | 1.13(1.08, 1.19) | <.001 |
| High | 1.54(1.11, 2.14) | .009 | 1.49(1.17, 1.97) | .027 | 1.23(1.11, 1.36) | <.001 |
| healthy lifestyle score |  |  |  |  |  |  |
| ≥3 | 1 (Reference) | - | 1 (Reference) | - | 1 (Reference) | - |
| 2 | 1.27(1.11, 1.46) | <.001 | 1.66(1.43, 1.93) | <.001 | 1.23(1.14, 1.32) | <.001 |
| 1 | 1.51(1.31, 1.74) | <.001 | 1.89(1.63, 2.18) | <.001 | 1.47(1.36, 1.58) | <.001 |
| 0 | 1.79(1.52, 2.11) | <.001 | 2.22(1.89, 2.61) | <.001 | 1.56(1.43, 1.71) | <.001 |

**^a^**High education:≥5years.

**^b^**Medium education: 1-5years.

**^c^**Low education:0years.

**^d^:**Hazard Ratio

## Table S15.Association of social stress, healthy lifestyle and mortality

| **Variable** | **Model 1^a^** | **Model 2^b^** |  |
| --- | --- | --- | --- |
|  | **HR(95%CI)^c^** | **HR(95%CI)^c^** | ***P-*value** |
| **Social stress** |  |  |  |
| Low | 1 (Reference) | 1 (Reference) |  |
| Medium | 1.15(1.10, 1.21) | 1.17(1.12, 1.24) | <.001 |
| High | 1.24(1.12,1.37) | 1.27(1.12,1.43) | <.001 |
| **Healthy lifestyle score** | 1 (Reference) | 1 (Reference) | - |
| ≥3 | 1.65(1.53, 1.78) | 1.29(1.20, 1.39) | <.001 |
| 2 | 1.52(1.42, 1.62) | 1.50(1.39, 1.61) | <.001 |
| 1 | 1.65(1.53, 1.78) | 1.65(1.52, 1.81) | <.001 |

**^a^**Model 1.Participants who died during the first year of follow-up were excluded.

**^b^**Model 2.Participants who died during the first 2 year of follow-up were excluded.

**^c^:**Hazard Ratio

## Table S16. The mediating role of lifestyle factors (smoking, alcohol consumption, physical activity, and diet score) in the relationship between social stress and all-cause mortality

|  | **Mediation analysis** | | | | | |
| --- | --- | --- | --- | --- | --- | --- |
|  | **P for Prop. Mediated** | **Prop. Mediated** | ***P* for ADE^a^** | **ADE^a^** | ***P* for ACME^b^** | **ACME^b^** |
| Drinking status | 0.74 | -0.0002(-0.005, 0.003) | <.001 | 0.041(0.029, 0.054) | .74 | 0.000(-0.0002, 0.0001) |
| Smoking status | 0.57 | -0.004(-0.023,0.012) | <.001 | 0.041(0.029,0.053) | .57 | -0.0002(-0.0001.0.0005) |
| Physical activity | <.001 | 0.091(0.060,0.146) | <.001 | 0.037(0.024,0.051) | <.001 | 0.004(0.003,0.005) |
| Dietary score | .004 | 0.018(0.005,0.037) | <.001 | 0.041(0.029,0.053) | .004 | 0.0001(0.0002,0.001) |

**^a^**:Average Direct Effect

**^b^**:Average Causal Mediation Effect

## Table S17. Association of SS with all-cause mortality after adjustment for weighted lifestyle score and other covariates

| **Variable** | **Case/N** | **HR(95%CI)^a^** | ***P-*value** |
| --- | --- | --- | --- |
| Social stress |  |  |  |
| Low | 3986/6981 | 1 (Reference) | - |
| Medium | 9425/11662 | 1.14(1.10, 1.19) | <.001 |
| High | 588/683 | 1.26(1.16, 1.38) | <.001 |
| Healthy lifestyle score |  |  |  |
| Healthy | 3446/6141 | 1 (Reference) | - |
| Intermediate | 5740/6744 | 1.38(1.31, 1.44) | <.001 |
| Unhealthy | 6362/4813 | 1.61(1.54, 1.68) | <.001 |

**^a^:**Hazard Ratio

## Table S18. Association of social stress, healthy lifestyle (reclassification) and mortality

| **Variable** | **Case/N** | **HR(95%CI)^a^** | ***P-*value** |
| --- | --- | --- | --- |
| Social stress |  |  |  |
| Low | 3986/6981 | 1 (Reference) | - |
| Medium | 9425/11662 | 1.15(1.11, 1.20) | <.001 |
| High | 588/683 | 1.29(1.18, 1.40) | <.001 |
| Healthy lifestyle score |  |  |  |
| ≥2 | 3446/6141 | 1 (Reference) | - |
| 1 | 5740/6744 | 1.26(1.32, 1.47) | <.001 |
| 0 | 6362/4813 | 1.39(1.22, 1.31) | <.001 |

**^a^:**Hazard Ratio

## Supplementary Figure 1. Impact of healthy lifestyle on all-cause mortality across different social stress group by age group


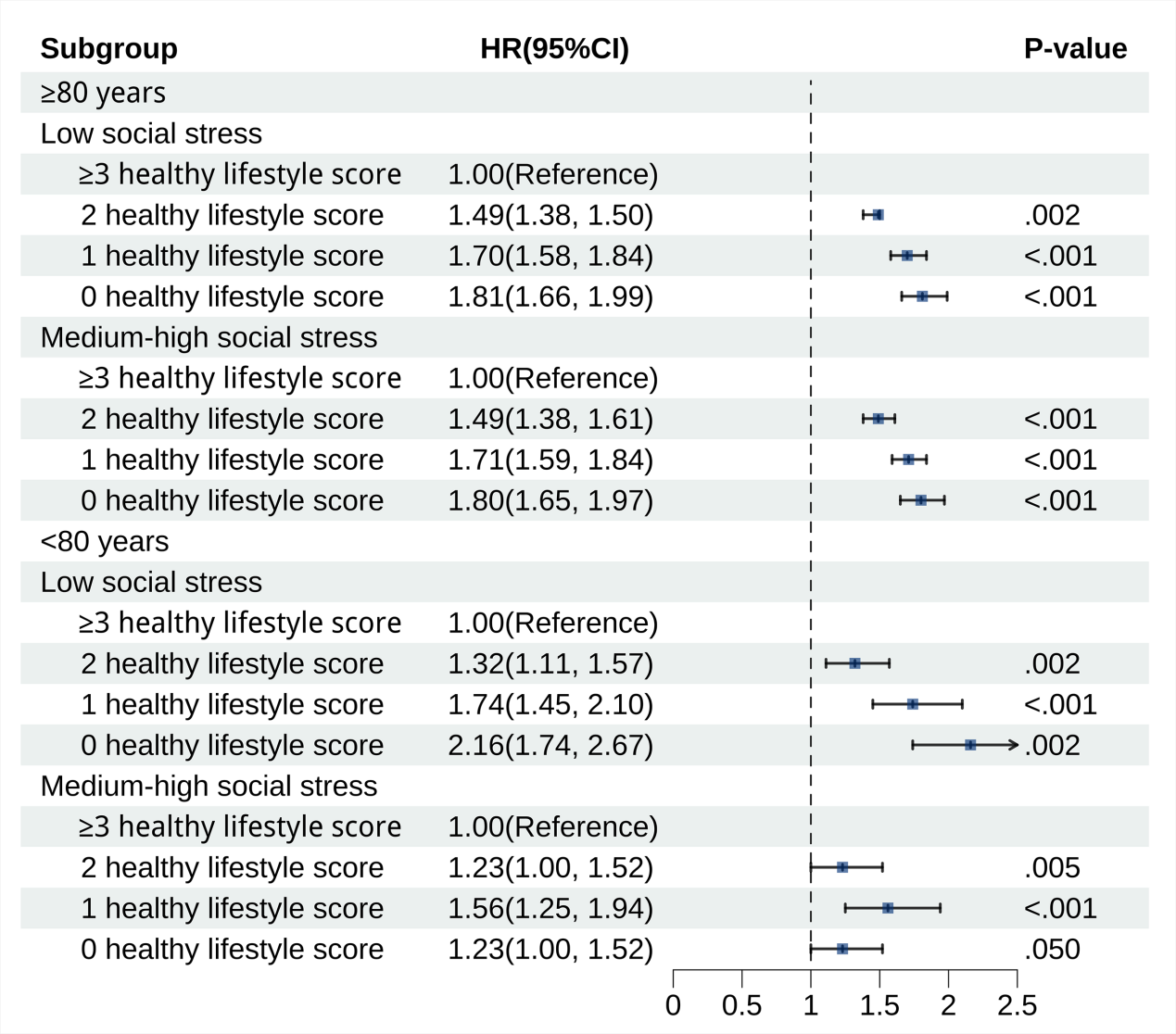


Note: All covariates were adjusted.
